# Improving treatment plan evaluation with automation

**DOI:** 10.1120/jacmp.v17i6.6322

**Published:** 2016-11-08

**Authors:** Elizabeth L. Covington, Xiaoping Chen, Kelly C. Younge, Choonik Lee, Martha M. Matuszak, Marc L. Kessler, Wayne Keranen, Eduardo Acosta, Ashley M. Dougherty, Stephanie E. Filpansick, Jean M. Moran

**Affiliations:** ^1^ Department of Radiation Oncology University of Michigan Ann Arbor MI; ^2^ Varian Medical Systems Palo Alto CA USA

**Keywords:** treatment planning, quality assurance, automation

## Abstract

The goal of this work is to evaluate the effectiveness of Plan‐Checker Tool (PCT) which was created to improve first‐time plan quality, reduce patient delays, increase the efficiency of our electronic workflow, and standardize and automate the physics plan review in the treatment planning system (TPS). PCT uses an application programming interface to check and compare data from the TPS and treatment management system (TMS). PCT includes a comprehensive checklist of automated and manual checks that are documented when performed by the user as part of a plan readiness check for treatment. Prior to and during PCT development, errors identified during the physics review and causes of patient treatment start delays were tracked to prioritize which checks should be automated. Nineteen of 33 checklist items were automated, with data extracted with PCT. There was a 60% reduction in the number of patient delays in the six months after PCT release. PCT was successfully implemented for use on all external beam treatment plans in our clinic. While the number of errors found during the physics check did not decrease, automation of checks increased visibility of errors during the physics check, which led to decreased patient delays. The methods used here can be applied to any TMS and TPS that allows queries of the database.

PACS number(s): 87.55.‐x, 87.55.N‐, 87.55.Qr, 87.55.tm, 89.20.Bb

## I. INTRODUCTION

In order to improve safety, radiation oncology departments have utilized checklists to standardize processes and increase compliance with departmental policies.^1^, ^2^, ^3^ Medical Physics Practice Guideline 4^(4)^ makes recommendations on the creation and use of checklists because of their value in improving safety. As an increasing number of processes are moved to an electronic workspace, the need for electronic checklists has risen.^5^, ^6^ This is especially true for the treatment planning process. It has been shown that the physics check of the treatment plan has the greatest effectiveness for catching grave errors.^(7)^ With a fully electronic process and record, checking treatment plans is complicated by the need to navigate multiple workspaces within the treatment planning system (TPS) and treatment management system (TMS). Tedious manual checks of plan parameters can lead the physicist to focus on the mundane details of electronic charting instead of overall plan quality, and increase the chances of missing serious errors.

The use of automation and safety barriers are more effective for safety and quality than implementing policies and procedures, which have been shown to be the most prevalent, but least effective, safety method for preventing errors.^(3)^ Since the physics plan evaluation requires checking similar plan parameters for each patient, this process is ideal for automation. Although several investigators have created and implemented programs to automate portions of the electronic plan check,^8^, ^9^, ^10^, ^11^, ^12^, ^13^, ^14^, ^15^ the clinical impact of such tools, as well as intended improvements in efficiency, have not been quantified.

Quality assurance of the treatment plan comprises many facets including a review of the dosimetric aspects of the plan. In addition to the dosimetric aspects, there are many parameters to review to ensure that the plan is deliverable and meets the departmental policies and guidelines. Our goal with PCT was to reduce the amount of time spent reviewing plan parameters that must be individually validated for each treatment (e.g., calculation model, field dose rate) and enable the physicist to have more time for evaluating dosimetric plan quality. In this work, first‐time plan quality refers to whether the treatment plan has been prepared according to the physician's intent and correctly prepared for treatment delivery. Plan quality was determined by comparing the treatment plan to the physician's written directive, as well as determining whether all departmental policies and procedures were followed for preparing the plan for treatment (e.g., naming convention, calculation model).

In order to improve standardization, first‐time quality, and efficiency of our initial physics plan checks, we have created a Plan‐Checker Tool (PCT) within the Eclipse Scripting Application Programming Interface (ESAPI)^(16)^ to automate portions of the physics pretreatment plan check as part of assessing the plan readiness for treatment. Data were collected at both the treatment unit and during physics checks to aid in the design of the tool, as well as to assess its effectiveness. PCT was designed as part of a departmental commitment to Lean Thinking^(17)^ to reduce waste while improving the efficiency and safety of workflows. PCT was intended to be run during multiple stages of the treatment planning process to catch errors early and prevent rework. Per the “consensus recommendations for incident learning database structures in radiation,”^(18)^ errors are defined as “failure to complete a planned action as intended or the use of an incorrect plan of action to achieve a given aim.” With respects to our data tracking, errors are any unintended action or omitted action in the treatment planning process that requires remediation during review of a plan by a physicist or causes a delay in the patient's treatment appointment. In this work, we describe the creation of Plan‐Checker Tool, its commissioning and implementation into the clinical workflow, and its subsequent clinical impact on improving first‐time treatment plan quality by quantifying the improvement in plan check efficiency and the reduction of both patient delays and treatment planning errors in our clinic.

## II. MATERIALS AND METHODS

Before PCT was created, data were collected in order to quantify the prevalence of mistakes or inconsistencies in treatment plans created by users in the TPS or TMS, referred to as TPS and TMS errors, and determine which could be mitigated by automation. For one year prior to the release of PCT, errors found during the physics plan check were documented by a subset of physicists. A spreadsheet was used to track the most common errors and provide feedback to software engineers about which checks should be automated. During this time, patient delays related to treatment planning and parameters in the TMS were also tracked. Any treatment that could not start at the scheduled treatment time due to a TPS or TMS error was defined as a delay. The most significant errors and those commonly found during physics checks, as well as those which led to patient delays, were prioritized for automation.

### A. Department software and workflow

External beam treatment planning was done with Eclipse (Version 11, Varian Medical Systems, Palo Alto, CA) and treatment delivery was managed with Aria (Version 11, Varian Medical Systems) during the time frame studied. The department uses the vendor‐supplied workflow tool known as the CarePath (Varian Medical Systems) to define different workflows which are part of the treatment planning and delivery process. The CarePath contains tasks which are assigned to either individuals (such as the attending physician) or groups (such as the physics group for plan and monitor unit (MU) checks). Physicians are responsible for approving treatment plans (represented by setting the plan status to “Reviewed”) and approving prescriptions in the vendor‐supplied “Prescribe Treatment” module. Plans and prescriptions are linked, meaning the prescription (created in the TMS) is attached to the plan as part of the department's policy. The design in this software version does not prevent inconsistent data between portions of the plan and prescription. For example, the TPS (Eclipse, v11, Varian Medical Systems) does not force that the manually entered prescription matches the parameters in the linked treatment plan. The prescription can be written for 6 MeV electrons and linked to a plan for 16 MV photons. There can also be a mismatch in dose per fraction and total dose. For each treatment fraction, therapists confirm that prescriptions are approved and linked to a treatment plan as part of the department's workflow. Guidance documents were created by the department's electronic chart team on topics such as naming conventions for plans and treatment fields, the plan approval process, scheduling, and so on.

### B. Data collection on errors found during the physics plan review

Error tracking was done by a subset of physicists who were responsible for setting the policies and procedures for physics plan reviews. During this period, all new treatment plan checks were assigned to one physicist per day and this physicist recorded all errors found during plan checks. Errors were compiled into a spreadsheet that recorded the date, patient name, and description of the error. Throughout approximately one year, a subset of physicists tracked plan and plan preparation compliance on 37 individual days when they were assigned to treatment plan checks. An average of 5 ± 4 errors was found per day, with a range of 1–20 errors being found per day.

Out of 182 errors, those occurring three or more times are shown in Table 1. Errors were then ranked as high, medium, or low priority for automation. Errors with high severity and/ or high frequency were ranked as high priority. Any error occurring more than 10 times was ranked as high priority, as well as any error that could potentially negatively impact the dose delivered during treatment (e.g., omission of bolus). Errors which were ranked as low or medium severity and/or occurred less than 10 times were assessed for the complexity of building the automation with the option of being kept as a manual check with automation deferred to a future release.

**Table 1:** Treatment planning errors found in the physics check listed by frequency. Errors were ranked high, medium or low priority for automation due to frequency, severity, and complexity of coding for those errors which occurred at least three times during the tracking period

*Error Category*	*Examples of Error*	*Error Frequency*	*Priority for Automation*
Secondary check software	• Plan not exported to software	33 (18%)	High
	• Reference point does not have location to calculate MUs		
Planning directive	• Inconsistencies between the planning directive and plan	25 (14%)	High
	• Violation of dose constraints without documented acknowledgement from physician		
	• Previous treatment not considered		
Mislabeled field name	• Field has incorrect name	13 (7%)	High
	• Field does not following naming convention		
Reference points	• Point tracking incorrect dose	12 (7%)	High
	• Dose limits are incorrect		
Scheduling	• Plan was scheduled for the incorrect number of fractions	12 (7%)	High
	• The plan was not scheduled		
Prescription	• Mismatch of energy, dose per fraction, or total dose between plan and prescription	11 (6%)	High
	• Prescription not linked to plan		
Naming convention	• Plan name or course name does not follow departmental naming convention	11 (6%)	High
Imaging templates	• Imaging templates not attached for fields	8 (4%)	Low
	• Incorrect templates used		
CarePath error	• Incorrect CarePath for the patient's treatment type	8 (4%)	Medium
Setup fields	• Improper set‐up fields created	8 (4%)	Medium
	• DRRs missing overlay or match anatomy		
Incorrect shifts from CT sim to treatment	• Shifts from CT reference to isocenter are missing or incorrectly entered	7 (4%)	Medium
Bolus	• Bolus was not linked to fields	7 (4%)	High
	• Bolus was not listed as a field accessory		
Jaws/MLCs	• Jaws are not closed to the MLCs	4 (2%)	High
	• MLCs are open under the jaws		
Tolerance table	• Tolerance table incorrect or missing for fields	4 (2%)	Low
Plan status	• Plan not ‘Reviewed’ by physician	3 (2%)	High
	• Plan not ‘Planning Approved’ by dosimetrist		

### C. Data collection on errors contributing to patient delays at the treatment unit

Since not all errors or discrepancies are caught during the physics check, we wanted to track patient delays at the treatment units due to TMS and TPS errors to determine which errors were missed to improve patient care. These delays were reported weekly by therapists to physicists and dosimetrists. Delays were investigated to determine the root causes of the delay and then were categorized accordingly. Delays were tracked over a period of six months prior to the release of PCT to aid with prioritizing the initial checks to be automated. Errors that overlapped with errors commonly found during the physics check were also prioritized for automation.

Over the six‐month period, 121 delays were reported with an average of two delays per day. Table 2 shows errors which led to delays which occurred at least three times, along with the frequency and priority for automation. The priority for automation was set using the same scale as for Table 1. In addition to those found during the physics plan check, additional errors that caused delays included incorrectly scheduled treatment machine, missing or incorrect image guidance document, and lack of clearance for patient setup. The top occurring error was in treatment machine scheduling, where patients were scheduled on a machine incompatible with the treatment type (e.g., electron treatment on a photon‐only machine).

**Table 2:** List of patients delays (in order of frequency) that occurred at least three times over the six‐month period before the release of PCT. Errors are ranked either high, medium or low for priority for automation due to error frequency and complexity of coding

*Error*	*Description*	*Error Frequency*	*Priority for Automation*
Treatment machine scheduling	• Patient scheduled on a machine that is not compatible with their treatment	19 (16%)	High
Prescription	• Wrong energy, dose or dose per fraction	18 (15%)	High
	• Not linked to plan		
Incorrect plan status	• Treatment plan isn't treatment approved	15 (12%)	High
Image guidance document incorrect or missing	• Document is not filled out	13 (11%)	High
	• Document contains errors		
Reference point	• Point tracking incorrect dose	8 (7%)	High
	• Dose limits are incorrect		
Mislabeled field name	• Treatment or imaging field names are incorrect	8 (7%)	High
Clearance issue	• Field does not clear	8 (7%)	High
Incorrect or missing shifts	• Move sheet for patient positioning not completed	6 (5%)	Medium
	• Shifts incorrect		
Imaging templates	• Templates not included	5 (4%)	Low
	• Incorrect template attached		
Setup fields	• Missing match anatomy	4 (3%)	Medium
	• Incorrect angle		
Bolus	• Tray bolus not attached to field or incorrectly attached	3 (2%)	High

### D. Prioritizing checkers for automation

In the first clinical release, 19 of 33 of items from a checklist developed and used by physicists in our clinic for performing a plan and monitor check were automated (Table 3). The data from both errors found during the physics check and treatment delays were used to guide which checks should be automated. Out of 12 high‐priority items, 6 were chosen for automation for the first clinical release: scheduled treatment machine, reference point value and dose limits, prescription, plan and course naming convention, bolus, and plan approval status. Some high‐priority items, such as checks of plan quality and MLC/jaw position, were deferred for automation at a later date and remained as a manual check. PCT is unable to search dynamic documents in the TMS, such as setup sheets and image guidance documents, to validate information. These checks are performed manually. Automated checks were also created for calculation model and field dose rates.

Failure to export plans to the secondary check software was mitigated by developing software to automatically export the plan once its status changes to ‘Planning Approved’.^(19)^ Other high‐priority items, such as checking uploaded documents in the TMS system (e.g., planning directive), cannot be checked with the PCT framework and remain manual checks. While mislabeled field names were a high‐frequency error, the severity is low; therefore, automation of this check was deferred as a future enhancement. Several low occurring errors, such as incorrect dose rate, were chosen for automation since they could be easily added and because of the impact on delivery accuracy, especially for IMRT and VMAT.

**Table 3:** Automated and manual checklist items in the first clinical release of PCT

*Type of Check*	*Item Checked*
Automated	CT dataset name
	Course name
	Number of courses created per day
	Plan name
	Plan normalization
	Dose calculation model
	Dose calculation settings
	Prescription energy matches plan
	Prescription dose and dose/fraction matches plan
	Prescription and plan dose matches reference point dose
	Dose limits match reference point dose
	Bolus
	Field dose rates
	Plan approval status
	DRRs created for all fields
	DRRs have overlays and match anatomy
	Plan labeling
	Scheduled machine
	Prescription linked to plan
Manual	Interpolation of structures
	Presence of stray contouring points
	Quality of image registration
	Field names
	Required documents present
	Fraction scheduling
	Gantry clearance
	User origin set correctly
	Isocenter for imaging and treatment fields match
	Beam energy/modality appropriate
	Plan quality
	Couch moves from the CT reference
	Plan exported to second check software
	Check billing (Dosimetry only)

### E. Software development

PCT is an in‐house developed C# program created as a script of the Varian ESAPI, with additional extensions for data that are not available within the API. The API extension allows data to be extracted directly from the TMS and TPS databases. For example, this was necessary to extract the reference point daily and total dose limits. PCT development was driven by two important features: flexibility and high performance. Flexibility is achieved by creating an Extensible Markup Language (XML)‐based reconfigurable framework, while high performance is achieved by the use of a parallel architecture.

#### E.1 XML‐based reconfigurable framework

**Figure 1:**
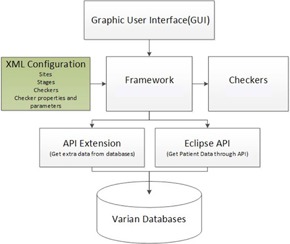
Schematic of the PCT architecture which includes a framework upon which checkers are built. Data can be accessed from multiple sources.

Architecturally, PCT consists of a framework and a set of independent check modules, called checkers, as shown in Fig. 1. An example checker would be one that compares the field dose rate to the departmental standard. Checkers are organized into stages to facilitate identification of errors at different stages of the treatment planning process. Based on the clinical workflow, we decided to organize the checkers into five stages: 1) prior to planning, 2) prior to MD review, 3) after MD created prescription, 4) prior to physics review, and 5) prior to treatment.

Stages are contained within a site, which is the highest level of the hierarchy. Although site is not meant to be analogous to body site, it was chosen with the design of supporting additional customization of PCT that could be specific to treatment modality and/or body site treatment (e.g., field‐in‐field breast, intracranial SRS). The version of PCT reported in this manuscript contains one “site” and is used for all treatment plans in our clinic. Both the framework and checkers are configured in a XML‐based configuration. The configuration controls dynamic loading .NET assembly, the checker types (automated or manual), and checker execution types (sequential or parallel). In some cases, it defines the checker‐expected values and patterns. As a result, it is easy to add or modify checkers. For example, our dose‐rate checker is automated with an expected value. The checker verifies that each treatment field has the default dose rate of 600 MU/min; therefore, the expected value for this checker is 600. This value could be modified if the default dose rate was changed. The framework facilitates upgrades to PCT as additional checklist items are added or automated. These features also enable PCT to be adapted by other clinics, such as our affiliated community clinics, by making minor modifications to checkers (e.g., updating expected values). The checker hierarchy, categories (sites, stages), and graphical user interface (GUI) layout are also easily modified due to the framework design.

#### E.2 Parallel architecture

We found that the execution time of running the individual checks in PCT sequentially was too long for the tool to be useful clinically (approximately 5 min per plan). To achieve acceptable performance, we have to run as many checks as possible in parallel to leverage the computer's multicore architecture. The Eclipse Scripting API, however, supports only single‐threaded operation, where a thread is a sequence of instructions that may execute in parallel with others. To achieve parallelism in execution of checks and acceptable performance, we adopted a strategy of extracting all information needed by the checkers up front in the main scripting thread and caching that information in an in‐memory XML document. Checks are then run in parallel on different threads, with each checker using the XML document as their data source instead of the Eclipse Scripting API. The cached in‐memory XML document is built up mainly from information extracted through the Eclipse Scripting API, via the ‘WriteXML methods for objects of interest; along with use of some API extensions we created to extract needed information that is not available on the Eclipse Scripting API. Using this method, we were able to reduce the PCT execution time from 5 min to approximately 1 min per plan.

#### E.3 PCT architecture

**Figure 2:**
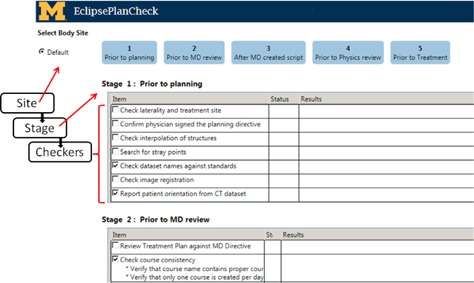
Example of the GUI of PCT with a schematic of the hierarchy added. The highest level of the hierarchy, the site, can be selected at the top left (this version has only one site, “Default”). The next level under site are the stages. In this version of PCT, there are 5 stages which are displayed in blue and the top of the display. All stages contain unique checkers (e.g., “Check dataset name against standards”) that are listed under the stage title (e.g., “Stage 1: Prior to planning”).

The XML configuration, the centerpiece of the architecture, feeds the control instructions to the framework (see Fig. 1). The framework controls the GUI, checker execution, direct database access through the API extension, and the patient data through the ESAPI. In other words, when PCT runs as a plug‐in in Eclipse, it loads the XML configuration file to get the hierarchy (sites, stages, and checkers), properties (sequential, parallel, check parameters), and parameters of all checkers. The configured hierarchy controls the GUI, shown in Fig. 2, and the configured checkers determine how to get the checker input data, how to run the checkers, and how to present the checker results. The API extension is called to supplement those checkers where the data within the ESAPI are incomplete or unavailable.

### F. Implementation

The checkers were created in a close collaboration between physicists and software engineers. Physicists provided detailed descriptions of what each checker was expected to validate within the TPS and TMS. After the checks were programmed, they were tested by physicists to ensure functionality. This was done by creating a test suite of anonymous clinical plans with errors intentionally introduced. An example of introduced errors is wrong calculation model or incorrect dose rate. Over 100 test plans were created with an average of 11 errors per plan. Several iterations of programming and testing were performed before each checker was finalized.

While some checkers were straightforward to program and to set pass/fail criteria, such as reporting and evaluating the dose rate for each field, others were more complex. For example, Table 4 shows the configuration and possible outcomes for our plan‐labeling checker. This check verifies that all plan names have been labeled according to their plan type following our clinical labeling policy. This check begins by looking at the MLC technique of each field in the MLC properties. Once the MLC technique is identified, the plan name is checked for the appropriate label and flagged if it is not found or if the label for another modality is found.

**Figure 3:**
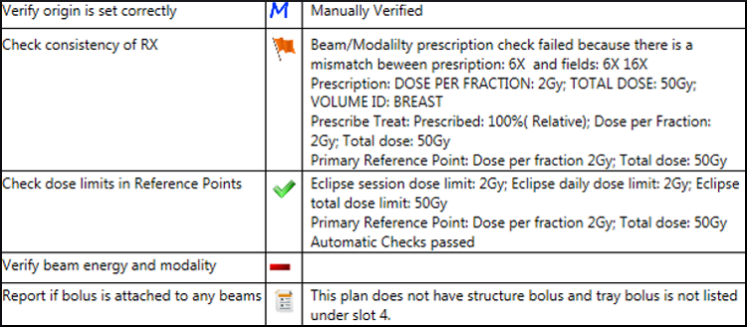
An example of flagged, passing, report, and manual checks. In this example, “Verify origin is set correctly” is a manual check that was toggled to “M” once manually verified. The “Check consistency of RX” is flagged due to a mismatch between the planned and prescribed energy. The green checkmark for “Check dose limits in Reference Points” indicates that the session dose limits match the planned reference point dose per fraction and total dose. “Verify beam energy and modality” is a manual check that has not been toggled. The bolus check displays a report symbol because structure and tray bolus were not found in the plan.

The labels for the automated checkers are: Pass, Flag, and Report. The checker outcome is displayed in the “Status” column by the corresponding icon. A green checkmark indicates that the automated check passed. An orange flag denotes that the checker information requires further review. The flag label was chosen over fail, because some flagged checkers may be acceptable. A report symbol indicates that information from the TPS or TMS database has been acquired and displayed for review. The user must read the results to determine if it is appropriate for the given treatment. For example, in Fig. 3, the report symbol lists that bolus is not attached to a field. The physicist would then determine whether or not that was correct for the treatment plan. The status symbols are shown in Fig. 3 for a subset of checkers. Since PCT contains 14 manual checklist items, icons were chosen that could be toggled once the manual check was completed. Manual check status icons are red dashes that can be toggled to an “M” when the check is completed. This enables the user to keep track of manual checks performed and avoid repeating checks if they are interrupted.

Our policy is that the final PCT report will only be saved by the physicist performing the plan check prior to treatment. Once the entire physics plan check has been completed, an upload button on the user interface is pushed and the PCT report is automatically uploaded as a document to the patient's record in the TMS via Varian's Document Service.^(20)^ This saves time and prevents the report from accidentally being uploaded to the wrong patient's record.

**Table 4:** Configuration of the plan‐labeling checker

*Plan Type*	*PCT Configuration*	*PCT will flag:*	*PCT will pass:*
3D	All beams must have MLC technique “Static” or “undefined”	If plan name contains “IM”, “VM”, “FIF”	If plan name does not contain “IM”, “VM”, “FIF”
FIF	One beam must have MLC technique “Dose Dynamic” with five or fewer control points	If plan name contains “IM”, “VM”	If plan name includes “FIF”
IMRT	One beam must have MLC technique “Dose Dynamic” with greater than 15 control points.	If plan name contains “VM”, “FIF”	If plan name includes “IM”
VMAT	Beams must have MLC technique “VMAT”	If plan name contains “IM”, “FIF”	If plan name includes “VM”

#### F.1 Plan‐Checker Tool commissioning

To commission PCT, clinical plans were made anonymous for testing. Known errors were introduced to these plans to validate the functionality of all the automated checkers. For example, prescriptions were created that intentionally mismatched the total dose or dose per fraction in the treatment plan. Over 100 plans were created with various errors so that each checker was validated against multiple plans with all possible outcomes (e.g., pass, flag, or report).

Once all checkers were verified to function properly using the test plans, PCT was used to check clinical plans. During the physics check, physicists would run PCT, and all reported values and checker statuses were verified by performing a manual check of each plan parameter in another window. This was done on over 100 clinical plans to verify functionality of all automated checkers. Any issues found resulted in feedback provided to the software engineers with detailed information on the error. After any revision of the script, the checkers were reevaluated with both the test plans and clinical plans.

#### F.2 Training

Before using PCT clinically, each user was trained by a physicist involved in the PCT project. Group training sessions were also held to demonstrate the proper use of PCT. Individually, each user had to run the script in the presence of the training physicist to demonstrate an understanding of how to properly use the tool. The automatically uploaded PCT report performed by each physicist was used to document training.

#### F.3 Integration into the treatment planning workflow

**Figure 4:**
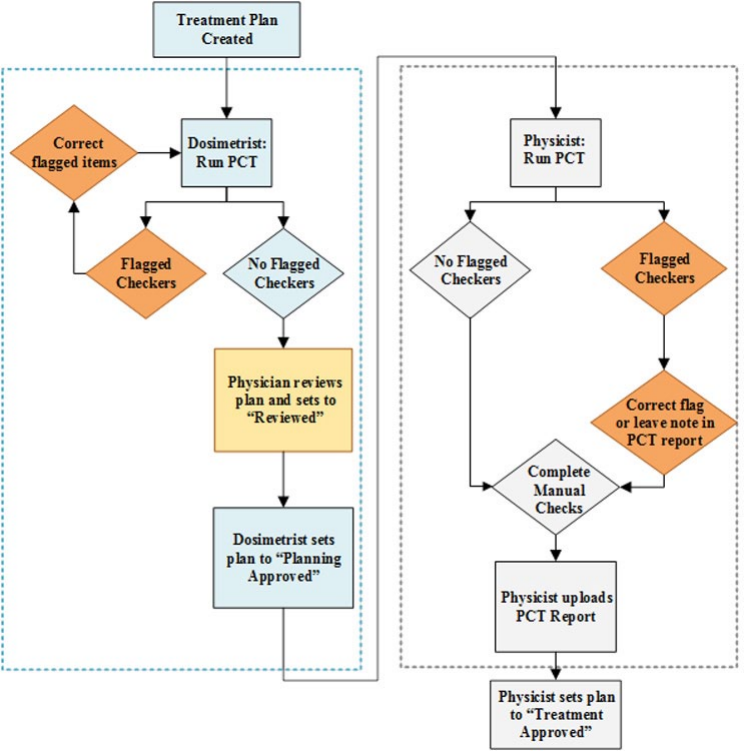
Treatment plan review workflow with Plan‐Checker Tool (PCT).

While use of PCT is encouraged at all stages of the planning process, our current policy is that PCT be run a minimum of two times: 1) by dosimetrists prior to physician review, and 2) by physicists during the plan and monitor unit (MU) review. A diagram of the treatment plan approval process with PCT is shown in Fig. 4. Prior to physician review, any flagged items found by PCT must be investigated and mitigated by the dosimetrist. At this time, dosimetrists are encouraged, but not mandated, to run PCT again before setting the plan to “Planning Approved”, to identify any errors associated with plan preparation such as creation of match anatomy layers on DRRs for patient localization at the treatment unit. When a physicist begins a plan and MU review, he/she is encouraged to immediately run PCT to look for any flagged items that need to be investigated. All flags are expected to be mitigated when possible. When a flag does not correspond to a plan error, a note must be added in PCT report explaining the flag. An example of an acceptable flag would be mismatch of daily and session dose limits due to a twice‐a‐day (BID) treatment.

## III. RESULTS

PCT was released on February 9, 2015. We then assessed whether or not there were any process improvements as measured by delays at the treatment units and errors found during the physics check. All patient delays related to TPS and TMS errors were compared for the six months prior to clinical release of PCT to delays in the six months post‐PCT release. Errors found during the physics plan review were also compared to assess any improvement in first‐time plan quality.

### A. Errors found during the physics plan review post‐PCT release

We continued to monitor plan quality by recording errors found during the physics plan review, now with the addition of PCT to the workflow. During the six months after release of PCT, errors were recorded on 44 unique days representing tracking by a subset of physicists. A total of 184 errors were recorded with an average of 4 ± 4 errors found per day, which is similar to the number of errors found per day prior to the release of PCT.

**Figure 5:**
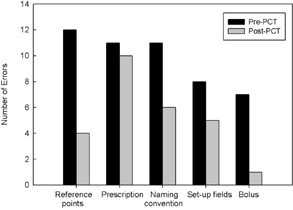
Comparison of errors found in the physics check before and after release of PCT with checkers that became automated.

A comparison of the top errors before and after PCT release is shown in Fig. 5. Reference dose errors found during the physics check were reduced by 67%. Errors related to bolus also decreased by over 70%. Errors related to prescriptions remained a high‐frequency error due to the manual entry of prescription parameters in the Prescribe Treatment workspace. The prescription parameters are transcribed from a dynamic document and used to create the electronic prescription. PCT is run before this step in the treatment planning process; therefore, more errors related to prescriptions are found during the physics plan review.

### B. Comparison of errors causing delays pre‐ and post‐PCT release

**Figure 6:**
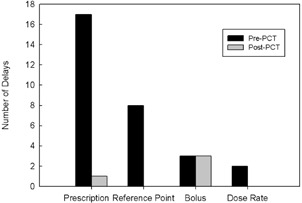
After release of PCT, a number of delays have been eliminated. One prescription delay occurred because PCT was not run on a simulation on‐set plan. Retraining was done to ensure all plans have a PCT report prior to treatment.

During the six months after release of PCT, there were 48 patient delays due to TMS and TPS errors which is a reduction of 60% when compared to the six months prior to the clinical release of PCT. Only four of these errors were attributed to errors that could have been caught by PCT, and three of those errors were related to bolus. There was one delay due to a prescription not being linked to the plan. In this case, PCT was not run on the plan due to it being a simulation–on‐set with no dose calculation in Eclipse where PCT is typically run. Retraining was completed to ensure that all plans have a PCT report prior to treatment. Figure 6 shows some of the most common causes of treatment delays and their corresponding frequency before and after PCT release. All other delays were due to errors in parameters that are either manually entered or checked, such as mislabeled field names or errors in manually created documents (e.g., image guidance and setup documents), or were related to workflow, such as the treatment plan not being ready on time or QA not completed on time.

### C. Efficiency improvements

To determine the improvement in efficiency of the physics treatment plan review, a time study was conducted. The PCT was found to load the results of the 19 automated checks with an average of 2.6 s. A subset of physicists was timed while performing the 19 checks manually using a paper checklist. The average time to complete the checks manually was 258 s, resulting in a time savings with PCT of approximately 255 s per plan. In the year after clinical release of the tool, 2830 PCT reports were created for clinical treatment plans; therefore, an estimated 200 hrs were saved by automating 19 checklist items. Also, before the release of PCT, a plan check was performed both before and after measurement‐based plan QA. Additional time savings were gained by the elimination of the second, post‐QA plan check. This step was eliminated because PCT documents all checks performed in the initial physics check. Accounting for this change in workflow, an additional 88 hrs were saved. The chart‐check process for the first weekly physics check after the start of treatment was changed to include a review of the PCT report rather than a complete review of the treatment plan. Further time savings have been reported from elimination of duplicate checks due to interruptions, although these savings were not quantified. Overall, an estimated 489 hrs were saved solely from the automation of 19 checklist items. See Table 5 for a detailed breakdown of time savings.

**Table 5:** Estimated time‐saving from automating 19 (of 33) checklist items in the physics plan check

*Activity*	*Number of Plans*	*Time Saved per Plan (min)*	*Total Time Saved (min)*
Plan check	2830	4.25	12028
IMRT/VMAT Plan check	1240	4.25	5270
First weekly chart check	2830	4.25	12028
Total time savings			29326 (488.8 hrs)

## IV. DISCUSSION

There have been numerous efforts in radiation oncology to automate QA of treatment plans. Li et al.^(11)^ developed QA software in an external program in C# to manage and assist with chart QA. Furhang et al.^(14)^ developed an Excel macro to guide the user through the chart‐check process. Work by Yang et al.^(5)^ used several different programming languages with over 300 MATLAB programs, including automatic comparison of the prescription and treatment plan. They also used a PDF and Microsoft Word parser to determine if required documents were completed and approved. The focus of our work was to standardize the plan‐check process and to make it more efficient and effective, such as by reducing the number of workspaces needed for plan checks. We chose to create PCT within the TPS. Using the ESAPI and API extensions, we were able to report all automated check results, along with our manual checklist items, on one screen within the TPS and to document additional relevant notes. Our workflow requires an additional open window of the TPS to verify plan quality and to investigate any flagged checks, but it does not require any external programs. Since data aren't exported from the TPS and TMS, we do not need to confirm any transfer of data. While PCT was developed for this work in a single‐vendor TPS/TMS environment, the concepts developed can be used for any TMS/TPS that supports data query. For example, a similar program, AutoLock,^(15)^ was written in Java for the Pinnacle TPS. To implement a program similar to PCT, we refer readers to their respective treatment planning systems user manual for information about database query through either scripting or in‐house developed software.

Comparison of the treatment plan to similar previously treated plans is another technique for assessing plan quality and catching gross errors. Kalet et al.^(21)^ have used Bayesian networks to determine whether plan parameters are within the normal scope of practice. Furhang et al.^(14)^ used both inter‐ and intraplan comparison to highlight outlier treatment plans. PCT is complementary to such broader checks and could be integrated with global evaluations of overall plan quality. For example, our clinic also has another API script under development for interplan comparison of VMAT complexity based on a modulation penalty that we had used previously during optimization.^(22)^

Like Breen and Zhang,^(13)^ automation of checks enabled us to catch errors made in the TPS and TMS more easily and to identify problems earlier in the planning process. This is evident by the elimination of several types of patient delays. Easy identification of minor problems, such as those that cause delays but would not render harm to patients, can allow the physicist to concentrate on overall plan quality.^(9)^ When compared to performing checks manually, automation of the 19 checklist items led to considerable time savings in our clinic. The electronic checklist also reduces rework after interruptions by documenting progress of the plan check. In addition to automation, PCT has resulted in increased standardization in our physics plan checks. Standardization has been a goal in several other automated plan‐check tools.^5^, ^11^, ^12^ Our electronic checklist and resulting PCT report has led to a standard review for every treatment plan. This ensures our ability to provide safe, quality care for all patients. Prior to the release of PCT, users had multiple checklists which they used to assist in the plan‐check process.

While approved checklists were stored on a shared drive, some users preferred to use a printed checklist which may not have always been up to date. With PCT, we ensure that the electronic checklist is current and the same for all users.

Overall, PCT led to an improvement in first‐time plan quality in all areas with automated checkers. The number of physics plan‐check errors identified per day remained constant, which we can attribute to two reasons. We believe that the use of automation has caused the errors to be more easily identified, so more of the errors are being caught during the physics checks. This was proven by the elimination of patient delays due to prescription errors and the mitigation of events, which may have previously reached the treatment units. Bolus errors have continued to cause patient delays despite automation. One delay was due to removal of bolus mid‐treatment, while the remaining two were caused by noncompliance with reading the reported bolus status within the PCT report. PCT is not required to be run again during plan revisions for minor changes in plan parameters, so PCT is not able to catch errors at this point in the treatment planning process. Physicists have now been advised to rerun PCT, in order to identify any errors that occurred during the plan revision (e.g., accidental removal of match anatomy, tray bolus status). In order to further address errors that continued to cause delays even after the implementation of PCT, a prestart checklist for therapists was created for high‐frequency delay items (e.g., prescription) and additional checks (e.g., field ordering, correct appointment time length). This checklist was added two months after the release of PCT and has also helped to reduce the number of treatment delays.

Incomplete compliance by dosimetrists in running the tool after planning and MD approval explains why some errors that are automated with PCT continue to be found routinely during the physics check. Strategies are under development for a future release of PCT to require a dosimetrist check with PCT prior to submission of the plan for review by a physicist. We are also planning to include the ability to have notes saved within the program from a previous user. This would enable the dosimetrist to leave notes in the report that would be visible to the physicist when the script is subsequently run on the same plan. This would facilitate better communication for flagged checks or variations in the plan that were approved by the physician but not indicated in the original planning directive. Department policy requires that the PCT report be reviewed during the first weekly chart check and when reviewing pretreatment measurement QA results for IMRT and VMAT patients. Throughout the release of PCT, data have been collected to prioritize new automatic checkers for future releases. Recent updates to PCT included automated checks for patient orientation, field tolerance tables, and the number of isocenters. Data tracking will continue to measure the impact of the newly released automated checks. Treatment planning technique (VMAT, SRS) specific checks have also been proposed for automation. This would eliminate the need for separate checklists for each treatment type. Due to its high frequency in plan checks and in patient delays, an automated check for field naming will also be implemented in the future. For CT dataset labeling, our process requires that the dataset include the year, month, and day in the name. The Radiation Oncology‐Incident Learning System (RO‐ILS) has reported a safety hazard where an old dataset was used for planning.^(23)^ The dataset checker will be enhanced in the future to flag if a dataset was created outside of a certain time range so that incorrect datasets can be flagged before a patient is treated. We regularly analyze events reported in our department's incident learning system and submit enhancement requests using a bug tracker software. As a result of this experience, automated checks of laterality in plans names and a more robust prescription checker are under development. Also, we have begun a failure modes and effects analysis (FMEA) on our treatment planning process to prioritize the next set of automated checks. The importance of analyzing experience with both incident learning and FMEA has been demonstrated to be invaluable.

We have successfully released PCT at several of our affiliated community practice clinics. Each clinic has a unique workflow and plan parameters (e.g., calculation model, naming conventions) demonstrating the flexible and robust design of the PCT framework. Configurable checkers can be modified by the physicist to support each clinic's workflow and needs. We have also been collaborating with other physicists in different practice environments on how to further improve the tool.

## V. CONCLUSIONS

PCT was created to streamline the physics plan review, improve first‐time plan quality, and decrease patient delays due to errors in treatment planning and preparation of the plan for treatment using a commercial TPS and TMS (Varian Medical Systems). We were able to automate 19 of 33 checklist items and measured a reduction in delays at the treatment unit. The use of automated checks led to the elimination of patient delays due to manually entered incorrect parameters in reference points and prescriptions. Compared to the six months prior to clinical release of PCT, there was a 60% reduction in patient delays in the six months after PCT release. While the number of errors found during the physics check did not decrease, automation of checkers increased visibility of errors during the physics check which led to decreased patient delays. The number of errors found during the physics check is expected to decrease as compliance with running the tool during treatment planning improves. The use of automation and an electronic checklist has made PCT a valuable tool for improving the workflow of electronic treatment plan checks and improving first‐time plan quality. We have successfully implemented it for use on all external beam treatment plans in our clinic.

## ACKNOWLEDGMENTS

The authors would like to acknowledge the contributions of Bonnie Durbin for collection of the patient delays data during her interim as Assistant Chief Therapist. The authors would also like to thank the therapists and physicists who participated in data collection. This work was partially supported under contract P01CA059827.

## COPYRIGHT

This work is licensed under a Creative Commons Attribution 3.0 Unported License.

## Supporting information

Supplementary Material

Supplementary Material
