# Supplementary figures and images for: Improving treatment plan evaluation with automation
Source: J Appl Clin Med Phys. 2016 Nov 8;17(6):16–31. doi: 10.1120/jacmp.v17i6.6322 (PMC5378447; doi:10.1120/jacmp.v17i6.6322)

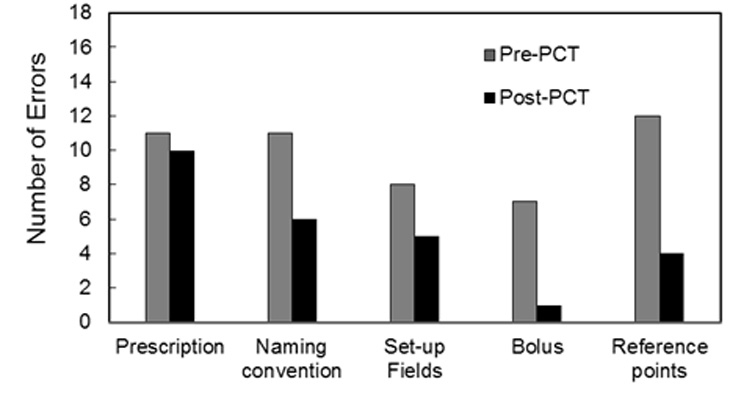

Supplement: Supplementary file 1 — Supplementary Material [file ACM2-17-016-s001.jpg]

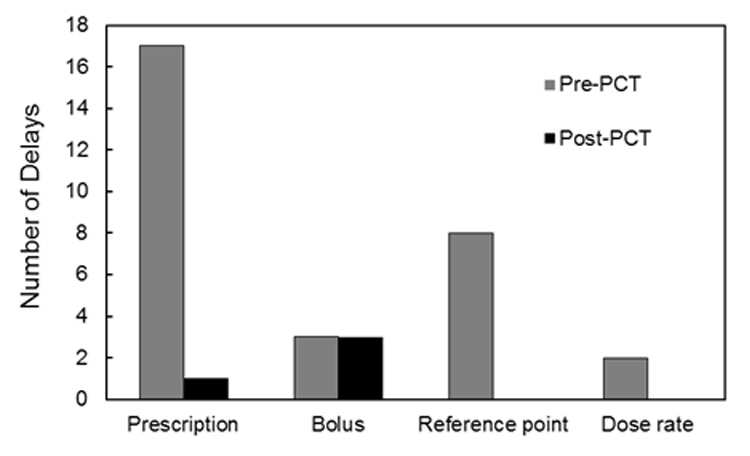

Supplement: Supplementary file 2 — Supplementary Material [file ACM2-17-016-s002.jpg]
